# The Serenity of the Meditating Mind: A Cross-Cultural Psychometric Study on a Two-Factor Higher Order Structure of Mindfulness, Its Effects, and Mechanisms Related to Mental Health among Experienced Meditators

**DOI:** 10.1371/journal.pone.0110192

**Published:** 2014-10-16

**Authors:** Ulrich S. Tran, Ausiàs Cebolla, Tobias M. Glück, Joaquim Soler, Javier Garcia-Campayo, Theresa von Moy

**Affiliations:** 1 University of Vienna, Vienna, Austria; 2 University Jaume I, Castelló de la Plana, Spain; 3 Ciber Fisiopatologia de la obesidad y la nutrición (CIBEROBN), Santiago de Compostela, Spain; 4 Department of Psychiatry, Hospital de la Santa Creu i Sant Pau, Barcelona, Spain; 5 Universitat Autònoma de Barcelona (UAB), Centro de Investigación Biomédica en Red de Salud Mental (CIBERSAM), Institut d'Investigació Biomèdica-Sant Pau (IIB-SANT PAU), Barcelona, Spain; 6 University of Zaragoza, Instituto Aragones de Ciencias de la Salud, Red Investigación Atención Primaria (RD 12/005/006), Zaragoza, Spain; University Hospital of Bellvitge-IDIBELL; CIBER Fisiopatología Obesidad y Nutrición (CIBERObn), Instituto Salud Carlos III; Department of Clinical Sciences, School of Medicine, University of Barcelona, Spain

## Abstract

**Objective:**

To investigate the psychometric and structural properties of the Five Facets Mindfulness Questionnaire (FFMQ) among meditators, to develop a short form, and to examine associations of mindfulness with mental health and the mechanisms of mindfulness.

**Methods:**

Two independent samples were used, a German (*n* = 891) and a Spanish (*n* = 393) meditator sample, practicing various meditation styles. Structural and psychometric properties of the FFMQ were investigated with multigroup confirmatory factor analysis and exploratory structural equation modeling. Associations with mental health and mechanisms of mindfulness were examined with path analysis.

**Results:**

The derived short form broadly matched a previous item selection in samples of non-meditators. Self-regulated Attention and Orientation to Experience governed the facets of mindfulness on a higher-order level. Higher-order factors of mindfulness and meditation experience were negatively associated with symptoms of depression and anxiety, and perceived stress. Decentering and nonattachment were the most salient mechanisms of mindfulness. Aspects of emotion regulation, bodily awareness, and nonattachment explained the effects of mindfulness on depression and anxiety.

**Conclusions:**

A two-component conceptualization for the FFMQ, and for the study of mindfulness as a psychological construct, is recommended for future research. Mechanisms of mindfulness need to be examined in intervention studies.

## Introduction

Converging evidence shows that mindfulness-based interventions (MBIs) are efficacious in the treatment of psychological disorders, the reduction of stress, and for improving well-being [1]. MBIs assume that mindfulness is an intrinsic state that all humans can cultivate through a variety of techniques, including, but not limited to, meditation. It is further assumed that long-term meditation practice cultivates mindfulness skills and that these skills, in turn, promote psychological well-being [2]. Parallel to the development of, and research on, MBIs there has been a growing interest regarding theoretical models of mindfulness [3], [4], its assessment [5], and its mechanisms of action [6].

Bishop et al. [3] proposed a two-component model of mindfulness that incorporates self-regulated attention (i.e., sustained attention on the present moment) and orientation to experience (i.e., the open, curious, and accepting attitude), which is one of the most widely-adopted in the field [7]. So far, however, only one of the many widely-used mindfulness questionnaires has been developed with direct reference to a two-component conceptualization, the Philadelphia Mindfulness Scale [8], [9]. Other widely-used questionnaires, like the Mindfulness Attention Awareness Scale (MAAS [10]), were developed with reference to Self-Determinant theory [11] or, as in the case of the Kentucky Inventory Mindfulness Skills (KIMS [12]), Dialectical Behavior Therapy [13].

One comprehensive and increasingly applied self-report measure of mindfulness, the Five Facets of Mindfulness Inventory (FFMQ [14]), was derived from scales that measure mindfulness as a trait in daily life. The FFMQ is composed of 39 items, stemming from the KIMS, the Freiburg Mindfulness Inventory [15], the Mindfulness Questionnaire [16], the MAAS, and the Cognitive and Affective Mindfulness Scale [17]. It has a five-facetted structure: Observe, Describe, Act with Awareness, Nonjudging of Inner Experience and Nonreactivity to Inner Experience. Besides in English, the FFMQ has been validated in a number of other languages (Chinese [18], Dutch [19], German [20], Spanish [21], and Swedish [22]), using patient, meditating, and non-meditating samples.

Even though conceptualized to represent a hierarchical factor structure, with mindfulness on top of the five facets, empirical research among non-meditators repeatedly showed that Observe fits only insufficiently into this structure, or is even unrelated to overall mindfulness [14], [19], [20], [22]–[24]. Among non-meditators, Observe appeared to indicate ruminative tendencies with regard to symptoms of anxiety, while facets of mindfulness were otherwise negatively related with psychological symptoms and positively with well-being [14], [20], [25]. Nonreact was reported to show only weak measurement properties [14], [20].

Recently [20], it was suggested that the FFMQ may be better represented by a two-factor higher-order structure, representing Self-regulated Attention and Orientation to Experience, as in the model of Bishop et al. [3]. It was predicted that these two factors, only modestly correlated among non-meditators [20], would be more strongly correlated among meditators, reflecting thus effects of meditation experience. Currently, data on two possible higher-order factors of mindfulness, their intercorrelation, and their associations with mental health among meditators are lacking. Moreover, various short forms of the FFMQ have been proposed [18], [20], [22], [26], indicating that its scale composition may need improvement. Items of the FFMQ were reported to show different psychometric properties among meditators and non-meditators [27]. Currently, it is unclear which FFMQ items are best suited to measure mindfulness, its facets, and its higher-order factor(s) among meditators.

Investigating the specificity of facets and higher-order factors of the FFMQ with regard to the beneficial effects of mindfulness on psychopathological symptoms and disorders appears specifically interesting for clinical research. Facet specificity of beneficial effects has been observed for symptoms of PTSD [28] and of anxiety and depression [29], involving specifically Actaware and Nonjudge that were later found to load also on the same higher-order factor, Orientation to Experience [20]. Changes of mindfulness may be reliably measured with the FFMQ, and the increase of mindfulness has been shown to specifically precede the reduction of stress, related to chronic illness, chronic pain, and other life circumstances, in a mindfulness-based stress reduction program [30]. More generally, it is known that mindfulness exerts beneficial effects on psychological health in various contexts and for different disorders [31], for example, by improving emotion regulation [32] and decreasing tendencies of rumination [33], [34], which play an important role in most psychopathologies [35], [36]. The evaluation of existing interventions, the development of new interventions, but also psychopathological research may thus benefit from investigations into mindfulness and by conceptualizing mindfulness on both a facet and on a higher-order level, using the FFMQ.

As to the mechanisms of action that underlie mindfulness, Hölzel et al. [6] proposed four major components: Attention regulation, body awareness, emotion regulation (reappraisal/revalorization; exposure, extinction, and reconsolidation), and a change in perspective on the self. Reviewed in detail by [6], mindfulness fosters the ability to sustain attention on a chosen object and to return attention to the object, when there have been distractions; bodily awareness is raised, as usually the object of attention is one of internal experience: Breathing, emotions, or bodily sensations. There are two effects on emotion regulation: One regarding the fostering of a nonjudgmental, accepting stance towards ongoing emotional reactions (‘reappraisal’ in [6]; here termed ‘revalorization’); the other regarding the fostering of a willingness to expose oneself to whatever is in the field of awareness, and to refrain from (automatic) internal reactivity. Lastly, mindfulness brings with it a change in perspective on the self: An adaptive detachment from identifying with a static self; instead, mindfulness fosters the awareness that the self is not permanent and unchanging.

Even though there is ample of empirical evidence with regard to these four mechanisms (see [6]; for more recent evidence see, e.g., [37], [38]), there have been, to our best of knowledge, no comprehensive attempts to systematically investigate the associations of these mechanisms with the facets and higher-order factor(s) of mindfulness among meditators yet. Determining which of these mechanisms primarily effectuate the beneficial effects of meditation and mindfulness on mental health could inform the future development of MBIs and help explaining why MBIs actually do work.

Therefore, the objectives of the present study were (a) to investigate the psychometric and structural properties of the FFMQ among experienced meditators, (b) to develop an abridged form of the FFMQ, thereby testing whether the item selections of previous short forms were replicable among meditators, and (c) to examine whether a two-factor higher-order structure, apparent among non-meditators, could also be observed among meditators. Finally, we examined the associations of the higher-order factor(s) of mindfulness with (d) depression, anxiety, and perceived stress, and (e) the proposed mechanisms of mindfulness, and we investigated (f) which mechanisms of mindfulness explained uniquely the beneficial effects of mindfulness on mental health (i.e., depression and anxiety). All psychometric and structural analyses, and analyses on the associations of mindfulness on depression and anxiety, were based on multigroup analyses, using two large and independent meditator samples from German-speaking countries and Spain. Results were thus cross-validated and analyses allowed also for cross-cultural comparisons, further broadening the generalizability of our results.

We hypothesized that a two-factor higher-order structure of mindfulness can be reliably replicated among experienced meditators, regardless of country of origin. However, we expected that the two higher-order factors were higher intercorrelated among meditators than previously among non-meditators. Furthermore, we expected that meditation experience increased the effects of mechanisms through which mindfulness exerts its beneficial effects on mental health, and that mechanisms of mindfulness fully mediated the associations of meditation experience and of mindfulness on mental health.

## Method

### Participants

Two meditator samples were used in this study, a predominantly German sample and a Spanish sample. Sample characteristics are displayed in Table 1. The German sample comprised 81% Germans, 15% Austrians, 2% Swiss, and 2% participants with other nationalities. Participants in both samples came from a wide age range and had various educational backgrounds. A majority meditated more often than on a weekly basis, around 52% four or more times a week in the German sample and around 36% on a daily basis in the Spanish sample (assessment of meditation frequency differed between samples). Most participants had meditated for more than four years, but there was also a wide range of meditation experience in the two samples.

**Table 1:** Sample Characteristics.

	German Sample	Spanish Sample	Statistical test
*n*	891	393	
Women, *n* (%)	661 (74.2%)	220 (56.0%)	?^2^(1) = 41.98***
Age, *M* (*SD*)	48.65 (10.52)	43.97 (10.60)	*t*(1280) = 7.30***, *d = *0.44
Min–Max	20–82	18–75	
Education			
Primary or lower secondary	10 (1.1%)	7 (1.8%)	?^2^(2) = 54.26***
Upper secondary	363 (40.7%)	77 (19.6%)	
University/diploma	518 (58.1%)	309 (78.6%)	
Frequency meditating			
> Once a week	675 (75.8%)	256 (66.1%)^a^	?^2^(2) = 13.46***
Once a week	66 (7.4%)	46 (11.9%)^ a^	
<Once a week	150 (16.8%)	85 (22.0%)^a^	
Meditation experience in years (*Md*)^b^	8.00	4.00	*U* test, *z* = 7.90***
Min–Max, interquartile range	0–45, 4–15	0–43, 2–10	
Meditation type^c^			
Zen	102 (11.5%)	110 (39.0%)	?^2^(4) = 369.32***
Vipassana	107 (12.0%)	136 (48.2%)	
Tibetan	54 (6.1%)	5 (1.8%)	
Yoga	348 (39.1%)	24 (8.5%)	
Other	279 (31.3%)	7 (2.5%)	

*Note*. ^a^ Based on 387 participants due to incomplete data. Based on ^b^ 738 and 348/^c^ 890 and 282 participants, respectively, for which data were available. *** *p*<.001.

Concerning sample differences, the German meditators included more women and were on average older and less highly educated than the Spanish meditators. However, the German meditators meditated more often and had on average more meditation experience than the Spanish meditators. With regard to meditation type, there were more Zen and Vipassana practitioners among the Spanish meditators, whereas more Tibetan meditation, yoga, and ‘other’ meditation styles practitioners among the German meditators. The ‘other’ category subsumed among German meditators mind-and-body meditation styles (15.8%), e.g., combinations of yoga and Vipassana or Zen, Transcendental Meditation (11.1%), Naikan (8.2%), Qi Gong (7.5%), Tai Chi (5.4%), and autogenic training (2.9%). The relatively large remaining proportion (49.1%) included various individual styles. Based on an Internet research, we concluded that from a technical point of view that these individual styles (e.g., Christian repetitive prayer or Tantra meditation practice) appeared not to differ substantially from styles like Vipassana, Zen, Transcendental meditation or yoga. They could also be roughly categorized into either concentrative, open monitoring, or a mixture of these two meditation styles.

The study was approved by the Aragon Ethics Committee (Spanish sample) and the department of psychology (German-speaking sample) and performed in accordance with the ethical standards of the 1964 Declaration of Helsinki (both samples) and Austrian ethical regulations for clinical research (German-speaking sample). All participants gave written informed consent prior to inclusion in the study.

### Procedure and measures

Participants in the German sample were recruited with help of (1) the German Buddhist Union (*Deutsche Buddhistische Union*, DBU), an umbrella organization of 63 member-groups comprising more than 600 German Buddhists groups and of individual Buddhists; (2) the professional institution for German Yoga teachers (*Berufsverband der Yogalehrenden in Deutschland*, BDY), numbering more than 3000 members; (3) the Austrian Buddhist Society (*Österreichische Buddhistische Religionsgemeinschaft*, ÖBR), an umbrella organization of around 30 Austrian Buddhist groups. DBU, BDY, and ÖBR disseminated detailed information on the study and the link to an online survey (www.sociosurvey.de) among their members and member-groups. Data collection itself took place between July and September 2011. Participation was entirely anonymous and participants received no remuneration. There were no exclusion criteria except that participants had to be over 18 years of age. As an incentive, participants could opt-in to take part in the drawing of six Amazon gift cards, worth € 50 each. Participants were also encouraged to disseminate the link to the online survey among meditating friends and acquaintances. Furthermore, the link was also disseminated among non-meditators; these data, however, were not of interest for the present study. The online survey was accessed 2099 times, and complete data of 984 persons (47%) were obtained, another 17 persons (<1%) provided incomplete data. Of the 984 persons with complete data, 891 (91%) were identified as practicing meditators and kept for further analysis. DBU, BDY, ÖBR, and participants disseminated the information on the study and the link to the survey independently and autonomously. Therefore, it is not possible to state exactly how many persons received the link to the survey and what percentage decided to participate.

The Spanish sample was recruited with help of several Spanish scientific research portals related to mindfulness and meditation. It was also sent to several mindfulness associations, Zen monasteries, and sanghas, and to a non-meditator convenience sample. A survey containing several questionnaires was developed using a commercial on-line survey system (www.surveymonkey.com; Portland, OR, USA), and a link to this website was posted on several other websites. The survey was available for response between April 2011 and December 2012. In total, 917 subjects accessed the link, while 850 (93%) voluntarily agreed to participate, of which 670 (79%) filled in the survey's scales and questionnaires fully. Of these, 384 (57%) were identified as practicing meditators and kept for further analysis.

#### Mindfulness

Mindfulness was assessed with the German [20] and the Spanish [21] form of the full 39-item FFMQ. Items are scored on a 5-point scale from 1 =  *never or very rarely true* to 5 =  *very often or always true*, assessing five facets of mindfulness, Observe, Describe, Acting with Awareness (Actaware), Nonjudging of Inner Experience (Nonjudge), and Nonreactivity to Inner Experience (Nonreact), with eight items (seven items in Nonreact) each. Cronbach alpha of the full facet scales lay between .79 (Observe) and .91 (Nonjudge) in the German sample and between .81 (Observe) and .94 (Nonjudge) in the Spanish sample, respectively; Cronbach alphas in Nonreact were satisfactory for both samples, .87 in the German and .86 in the Spanish sample.

#### Depression and anxiety

Symptoms of depression and anxiety were assessed with the depression and anxiety scales of the Brief Symptom Inventory (BSI; German form [39]) in the German sample, and the Depression, Anxiety and Stress scale (DAS-21 [40]; Spanish form [41]) in the Spanish sample. The BSI assesses the prevalence and distress caused by a variety of symptoms during the last seven days. Depression and anxiety are assessed with six items each, scored on a 5-point scale from 0 =  *not at all* to 4 =  *extremely*. The DAS-21 has three subscales that measure depression, anxiety, and stress with seven items each. Subjects assess the frequency/severity of 21 negative emotional symptoms during the previous week. Each item comprises a statement and four short response options to reflect severity and scored from 0 =  d*id not apply to me at all* to 3 =  a*pplied to me very much, or most of the time*. Cronbach alpha was.81 (depression) and.80 (anxiety) in the German sample and.90 and.85, respectively, in the Spanish sample.

For multigroup analysis, scores of the two samples were equated, using methods described by [42]. Equating allows the mapping of scores of different groups or measures on a common scale, ensuring that scores relate to the same underlying dimension. For equating, we identified similar items that had been devised in both groups. Items 10, 13, 16, and 17 of the DAS-21 depression scale were similar in content to Items 5, 2, 6, and 3 of the BSI depression scale, assessing feelings of hopelessness, depressed mood, lack of interest, and feelings of worthlessness (in this order); Items 7, 15, and 20 of the DAS-21 anxiety scale were similar in content to Items 10, 11, and 9 of the BSI anxiety scale, assessing tremulousness, fits of panic and fear, and being scared without reason (in this order). These sets of similar items were used for equating, utilizing structural equation modeling (see the Statistical analysis section).

#### Perceived stress

Perceived stress was assessed in the German sample with the German form the revised Perceived Stress Questionnaire (PSQ [43]), an economic and well-validated 20-item measure with four scales: Stress, Worries, Tension, Joy, and Demands, assessed with five items each. Items are scored on a 4-point scale from 1 =  *almost never* to 4 =  *usually*, asking respondents to rate the frequency of having experienced various stress reactions (Worries, Tension, Joy) and stressors (Demands) during the last four weeks. Cronbach alpha lay between.86 and.87 in the four scales.

#### Mechanisms of mindfulness

A number of likely mechanisms of mindfulness [6], were assessed in the Spanish sample with the Effortful Control Scale (EC [44]; Spanish form [45]), the Scale of Body Connection (SBC [46]; Spanish form [47]), the Experience Questionnaire (EQ [48]; Spanish form [2]), the Difficulties in Emotion Regulation Scale (DERS [49]; Spanish form [50]), and the Nonattachment Scale (NAS [51]).

The 19-item EC measured executive attention with three subscales: Attentional Control (seven items; the ability to intentionally shift and focus attention), Inhibitory Control (five items; the ability to inhibit a dominant, positively toned, response that may entail approach behavior), and Activation Control (seven items; the ability to suppress a dominant, negatively toned, response that may entail avoidance behavior). Items are rated on a 7-point Likert scale (1 =  *extremely untrue of you* to 7 =  *extremely true of you*). Cronbach alpha was .76.

The 20-item SBC measured Body Awareness (12 items; conscious attention to sensory signals that indicate the state of the body, i.e., tension, nervousness, relaxation) and Body Dissociation (eight items; body connection and separation, including emotional connection with one's body). Items are scored on a 5-point Likert scale, ranging from 0 =  *not at all* to 4 =  *all the time*. Cronbach alpha was.86 (Body Awareness) and.67 (Body Dissociation). For ease of interpretation, the Body Dissociation scale was reverse scored and termed Body Association (higher scores indicating higher bodily association) for purposes of the present study.

Aspects of emotion regulation were measured with the EQ and the DERS. The 11-item EQ measures decentering, i.e., the capacity to observe one's thoughts and feelings as temporary and objective events of the mind [2], [48]. Decentering is a unidimensional construct, related to metacognitive awareness [52], and comprises three facets: The ability to view one's self as not synonymous with one's thoughts, the ability not to habitually react to one's negative experiences, and the capacity for self-compassion (i.e., a positive and caring stance towards the self). Given that decentering is the ability of not being entangled with emotions and other internal events [48], it has been considered an aspect of emotional regulation in the present study. Items are rated on a 5-point Likert scale ranging from 1 =  *never or very rarely true* to 5 =  *very often or always true*. Cronbach alpha was .89.

The DERS measured difficulties in emotion regulation. The Spanish form [50], that was utilized in this study, comprises 28 items and five scales, instead of the 36 items and six scales of the English form (Non acceptance of Emotions, Difficulties Engaging in Goal-Directed Behavior When Distressed, Impulse Control Difficulties, Lack of Emotional Awareness, Limited Access to Emotion Regulation Strategies, Lack of Emotional Clarity; items of the Impulse Control Difficulties and Limited Access to Emotion Regulation Strategies scales form a single scale in the Spanish form). For ease of interpretation, the DERS scales were reverse scored in the present study so that higher scores reflected greater ability, not more problems with emotion regulation. We termed the scales in the present study in the following way: Acceptance of Emotions (seven items), Goals (four items), Control & Regulation (nine items; containing items of the Impulse Control Difficulties and Limited Access to Emotion Regulation Strategies scales), Emotional Awareness (four items), and Emotional Clarity (four items). With regard to their theoretical status, see [6], Acceptance of Emotions, Emotional Awareness, and Emotional Clarity each tap into the revalorization dimension of emotion regulation, whereas Goals and Control & Regulation into the exposure and reconsolidation dimension. Items are rated on a 5-point scale from 1 =  *almost never* to 5 =  *almost always*. Internal consistency in the present sample was .89.

Changes in perspective on the self were measured with the 30-item NAS. The NAS measures nonattachment, i.e., a subjective quality characterized by a relative absence of fixation on ideas, images, or sensory objects, as well as an absence of internal pressure to get, hold, avoid, or change circumstances or experiences [51]. Items are scored on a 6-point scale from 1 =  *disagree strongly* to 6  =  *agree strongly*. Cronbach alpha was .92.

### Statistical analysis

#### Structural analysis of the FFMQ and construction of a short form

The structure and measurement characteristics of the full FFMQ were assessed with multigroup structural equation modeling (SEM) in the two samples, testing for configural invariance (i.e., equality of the factor structure across the samples) of a correlated five-factor model [14].

A short form was then constructed, informed also by the results of a multigroup exploratory structural equation modeling (ESEM [53]) analysis that tested for item cross-loadings that may otherwise be overlooked in traditional SEM analysis. ESEM integrates exploratory (EFA) and confirmatory factor analysis (CFA) and allows the free estimation of cross-loadings as in EFA, but also provides standard errors and goodness-of-fit statistics as in CFA and SEM. ESEM was previously used to investigate the higher-order structure of the FFMQ in samples of non-meditators [20], but not the multidimensional structure of the items themselves. Items were then selected that: (1) loaded highest on their designated factor; (2) had no cross-loadings on the other factors; (3) were not redundant with other items in their scale; (4) maximized fit of a one-factor model (unidimensionality of facets) in both samples.

Structural invariance was re-assessed with the short form, testing: (a) configural invariance; (b) (full) measurement invariance (i.e., constraining all item loadings and thresholds to equality across samples); (c) partial measurement invariance, relaxing equality constraints for items where indicated by modification indices. The analyses (b) and (c) served to examine whether items in the FFMQ had the same measurement properties in the two samples and to determine a common origin and scale for estimating mindfulness in the two samples. Improvement of data fit of the partial measurement model versus the full measurement model was investigated with Mplus's DIFFTEST option.

Structural analyses were conducted with Mplus 6.11 [54], using the weighted least square mean- and variance-adjusted (WLSMV) estimator that is based on the polychoric item correlation matrix and that provides estimates of item loadings and thresholds. WLSMV estimation is suited for ordered categorical variables and provides robust parameter estimates, standard errors, and tests of model fit [55]. WLSMV estimation was previously used in FFMQ item analyses in samples of non-meditators [20]. Model fit was assessed using widely-used benchmarks [56], utilizing the comparative fit index (CFI), the Tucker-Lewis index (TLI; CFI and TLI: good fit: ≥.95, acceptable fit: ≥.90), and the root mean square error of approximation (RMSEA; good fit: *<*.06, acceptable fit: *<*.08). In models with small degrees of freedom (*df*), as in the ESEM analyses and analyses of unidimensionality of facets, model fit was primarily assessed with CFI and TLI as RMSEA may then be overinflated [57].

#### Higher-order structure of mindfulness

ESEM analysis currently does not allow the direct investigation of higher-order structures in item analysis; hence, facet factor scores of the final partial measurement invariance model were used to investigate the higher-order structure of the FFMQ with multigroup ESEM analysis, fitting a two-factor model to the data and using QUARTIMAX rotation and a maximum likelihood estimator that approximates the standard errors by first-order derivatives (MLF) [20].

#### Effects and mechanisms of mindfulness

Higher-order factor scores were then utilized in multigroup path models to investigate the effects of mindfulness on depression and anxiety, on perceived stress (German meditators only), on mechanisms of mindfulness (Spanish meditators only), and of mechanisms of mindfulness on depression and anxiety (Spanish meditators only), controlling also for meditation experience in each analysis. As meditation experience was positively skewed (skewness = 1.41 across both samples), the logarithm of meditation experience in months was used for all correlational analyses. Age and meditation experience correlated with *r* = .41 (*p*<.001) across both samples. In the presence of meditation experience, age exerted no significant (*p*>.05) or substantial effect (standardized parameter estimates <.10) on the other variables in any of the investigated models and was therefore excluded from all path analyses. Mplus 6.11 was used for path analyses, using the robust maximum likelihood (MLR) estimator that allows and corrects for non-normality in continuous scores and provides robust standard errors, based on a sandwich estimator. Mplus' full-information maximum likelihood (FIML) option was utilized, using all available data for parameter estimation, allowing the inclusion of participants with missing data regarding their meditation experience (see Table 1). Participants with missing data in meditation experience differed neither with regard to sex (χ^2^(1)  =  0.04, *p*  = .849), age (*t*(1280)  =  0.08, *p* = .938), nor education (χ^2^(2)  =  1.76, *p* = .414) from other participants, but reported a lower frequency of meditation than other participants (χ^2^(2)  =  758.45, *p*<.001). Thus, participants with missingness regarding their meditation experience were likely less experienced meditators.

#### Equating depression and anxiety scores for multigroup analysis

Items with similar content in the DAS-21 and BSI were used to equate factor scores of depression and anxiety in the two samples. Multigroup 1-factor models were fit to the data, constraining in a first step the loadings of the equated items to equality across groups (full measurement invariance), but relaxing these constraints in subsequent steps (partial measurement invariance), where necessary. Mplus 6.11 was used, utilizing WLSMV estimation. The BSI is scored on a 5-point scale, the DAS-21 on a 4-point scale; thus, the two highest response options were combined in the BSI items. Factor scores on a common scale were then used in multigroup analysis to investigate the effects of mindfulness on depression and anxiety.

## Results

### Construction of a short form and invariance testing

Fit of the configural invariance five-factor model in the full FFMQ is displayed in Table 2. While RMSEA appeared acceptable (<.06), CFI and TLI were below a cutoff of.95 each, indicating that fit could be still improved. A five-factor multigroup ESEM model fitted the data considerably better than the confirmatory model, χ^2^(1394) = 2904.15, CFI  = .971, TLI  = .969, RMSEA  = .041 [.039,.043]. Factor loadings are displayed in Table S1. Some items in Describe (Items 12 and 16) and Actaware (Items 23, 28, 34, and 38) displayed non-negligible cross-loadings on the Observe factor, while loadings of items in Observe where overall low (lowest in Items 11 and 36) and items had also cross-loadings on the Nonreact factor (Items 1 and 36). Item 13 in Actaware had a cross-loading on the Nonreact factor as well.

**Table 2:** Fit of Five-Factor Multigroup Models on All Five Facets.

	?^2^ (*df*)	CFI	TLI	RMSEA [90%-CI]
*Full FFMQ*				
Configural invariance	4130.30 (1384)	.947	.944	.056 [.054,.058]
*20-Item Short Form*				
Configural invariance	1028.76 (320)	.971	.965	.059 [.055,.063]
Measurement invariance	1400.26 (410)	.959	.962	.061 [.058,.065]
Partial measurement invariance*	1167.52 (385)	.968	.968	.056 [.053,.060]

*Note*. * Estimating loadings and thresholds of Items 4, 17, 18, 20, and 32 freely in both groups.

Final item selections and Cronbach alpha coefficients of the short scales are displayed in Table 3. Elimination of items improved unidimensionality of all five facets, strongest in Observe, Describe, and Actaware with regard to CFI and TLI (Table 4). Despite a cross-loading on the Nonreact factor, Item 13 was kept in Actaware as it had the highest loading of all items on its designated factor. Item selections were fully concordant with a previous item selection in samples of non-meditators [20] in Observe and Actaware, and partially concordant (three items) in Nonjudge. In Describe only one item (Item 32) was concordant, with Item 2 being similar in content to Item 37 that was selected in non-meditators instead. Largest differences were observed in Nonreact, where only the selection of one item (Item 21) matched across studies. Differences in item selections likely stemmed from differences in meditation experience, especially with regard to Nonreact that was found to be psychometrically weak among non-meditators [20]. Differences, however, may have also stemmed from differences in methodology, as the item selection in the present study also controlled for, and minimized, item cross-loadings via ESEM analysis.

**Table 3:** Factor Loadings in the Partial Measurement Invariance Five-Factor Model.

Observe			Describe			Actaware			Nonjudge			Nonreact		
Item	Ger	Span	Item	Ger	Span	Item	Ger	Span	Item	Ger	Span	Item	Ger	Span
**15**	.82	.78	**2**	.81	.85	**5R**	.81	.84	17R*	.74	.82	4*	.63	.75
20*	.49	.74	7	.81	.85	**8R**	.74	.78	**25R**	.90	.90	**21**	.75	.71
**26**	.81	.76	27	.79	.83	**13R**	.87	.91	**30R**	.90	.90	29	.79	.74
**31**	.73	.69	32*	.68	.48	18R*	.76	.85	**35R**	.83	.83	33	.87	.82
α	.75	.77		.81	.80		.83	.86		.87	.89		.80	.79

*Note*. Ger  =  German sample; Span  =  Spanish sample. α  =  Cronbach Alpha. Numbers refer to standardized factor loadings in the partial measurement invariance model where unstandardized loadings and thresholds were constrained to equality across samples. Items that were similarly retained in non-meditating samples (see text) are printed boldface. * Loadings and thresholds estimated freely in both groups. All *p*s<.001.

**Table 4:** Fit of One-Factor Multigroup Models in Individual Facets.

	?^2^ (*df*)	CFI	TLI	RMSEA [90%-CI]
Observe	263.48 (40)	.959	.943	.093 [.083,.104]
Observe–short	11.48 (4)	.998	.993	.054 [.019,.092]
Describe	680.16 (40)	.965	.950	.158 [.148,.168]
Describe–short	6.60 (4)	1.000	.999	.032 [.000,.073]
Actaware	867.30 (40)	.941	.918	.179 [.169,.190]
Actaware–short	39.64 (4)	.995	.986	.118 [.086,.152]
Nonjudge	225.08 (40)	.991	.988	.085 [.074,.096]
Nonjudge–short	18.79 (4)	.999	.996	.076 [.044,.112]
Nonreact	232.85 (28)	.982	.973	.107 [.094,.120]
Nonreact–short	10.50 (4)	.999	.996	.050 [.013,.089]

The configural invariance five-factor model fitted considerably better to the short form than to the full FFMQ (Table 2). Descriptively, a full measurement invariance model fitted the data as well. However, a partial measurement invariance model, estimating loadings and thresholds of five items, one per scale, freely across samples, improved the model fit significantly (DIFFTEST: χ^2^(25)  =  249.78, *p*<.001).

Table 3 displays standardized loadings in the final partial measurement invariance model. Even though unidimensionality was not violated in the respective scales in either sample, Item 20 had only a low loading in the German sample, whereas Item 32 in the Spanish sample, indicating differences in psychometric properties across samples/languages even in this purified FFMQ item selection. Based on estimates derived from the partial measurement model, samples differed significantly in three of the five FFMQ facets: The German meditators had slightly lower values in Observe (*d* = -0.15, *p* = .028), but higher values in Actaware (*d* = 0.44, *p*<.001) and Nonjudge (*d* = 0.21, *p* = .003) than the Spanish meditators (Describe: *d* = -0.06, *p* = .363; Nonreact: *d* = 0.07, *p* = .305).

Table 5 displays factor intercorrelations in the unconstrained configural invariance five-factor model in the full FFMQ and the final partial measurement invariance model in the short form. As can be seen, the factor intercorrelation pattern was similar in the two samples and was closely reproduced in the short form; however, consistent with observed cross-loadings, correlations of Actaware with Observe and Describe, and of Nonreact with Observe, were somewhat higher in the full FFMQ than in the short form. As items in the short form were free of cross-loadings, one may therefore infer that factor intercorrelations were more accurately estimated in the short form.

**Table 5:** Factor Intercorrelations in the Five-Factor Multigroup Models.

	Observe	Describe	Actaware	Nonjudge	Nonreact
Observe		.50/.48	.50/.48	.37/.33	.64/.71
Describe	.49/.47		.45/.43	.40/.44	.51/.51
Actaware	.35/.39	.33/.28		.61/.55	.54/.49
Nonjudge	.35/.32	.34/.33	.54/.54		.53/.55
Nonreact	.50/.66	.47/.56	.50/.42	.51/.53	

*Note*. Figures in the upper triangular matrix display factor intercorrelations in the configural invariance model in the full FFMQ (left: German sample; right: Spanish sample), whereas figures in the lower triangular matrix factor intercorrelations in the partial measurement invariance model in the short form. All *p*s<.001.

### Higher-order structure

Multigroup ESEM analysis of the facet factor scores indicated that the fit of a two-factor higher-order model could be still improved, χ^2^(11)  = 126.26, CFI = .956, TLI = .920, RMSEA = .128 [.108,.148]. Modification indices suggested a residual dependence of Observe and Describe. Allowing for this dependence, and constraining the variance of Nonreact to be positive, to ensure a positive definite correlation matrix, model fit was improved, χ^2^(10) = 88.43, CFI = .970, TLI = .940, RMSEA = .111 [.090,.132].

Facet factor loadings of this final model are displayed in Table 6. Residual variances of Observe and Describe correlated with *r* = .36 (*p*<.001) in the German and *r* = .13 (*p* = .018) in the Spanish sample. The loading pattern was broadly similar to the pattern in non-meditating samples [20]; however, Nonreact, not Observe, appeared to be the most important facet of Self-regulated Attention. In turn, Nonreact did not load on Orientation to Experience as among non-meditators. Nonjudge had a small positive loading on Self-regulated Attention. Similar to previous results, Actaware was the most important facet of Orientation to Experience, followed by Nonjudge. Observe had a small, but positive (negative in [20]), loading on this higher-order factor as well. The two higher-order factors correlated with *r* = .69 and.62 (*p*s<.001), respectively, considerably higher than in non-meditating samples [20].

**Table 6:** Facet Factor Loadings in the Two-Factor Higher-Order Multigroup ESEM Analysis.

	Self-regulated Attention		Orientation to Experience	
	German	Spanish	German	Spanish
Observe	.57***	.60***	.12*	.14*
Describe	.55***	.54***	.07	.08
Actaware	-.03	-.03	.90***	.89***
Nonjudge	.27***	.25***	.50***	.51***
Nonreact	.99***	.98***	-.03	-.03

*Note*. * *p*<.05, *** *p*<.001.

Based on estimates derived from this model, the German meditators had, with a medium effect size, higher scores than the Spanish meditators in Orientation to Experience (*d* = 0.53, *p*<.001), but not in Self-regulated Attention (*d* = -0.06, *p* = .316).

### Effects of meditation experience and mindfulness

#### Depression and anxiety

Clinically relevant levels of depression and anxiety in the German sample were similar to levels expected in the general population, according to the norm data of the BSI: With regard to depression 19.9% of participants, and with regard to anxiety 13.6% participants had a T value (adult norm of the BSI) at or greater than 63, considered reliably indicative of clinically relevant symptoms. This compares to around 16% that would have been expected with regard to normative data. In the Spanish sample 6.5% of participants reported severe or extremely severe symptoms in depression, whereas 7.1% in anxiety, according to the scoring of the DAS-21. Structural equation modeling revealed a good fit of a 1-factor model with full measurement invariance to the depression ratings of the German and Spanish meditators on the four common items of the BSI and DAS-21, χ^2^(18)  = 91.80, *p*<.001, CFI = .979, TLI = .986, RMSEA = .081 [.065,.097]. Relaxing equality assumptions for Item pair 7 (DAS-21) and 10 (BSI), fit of a 1-factor model with partial measurement invariance was also good for the three common anxiety items, χ^2^(6)  = 24.21, *p* <.001, CFI  = .986, TLI  = .986, RMSEA  = .069 [.042,.099]. A path model was fitted on the equated factor scores to explain the associations of the higher-order factors with depression and anxiety and of meditation experience on mindfulness (see Table S2 for the correlational pattern in raw scores). Equated factor scores correlated.91 and.84 (both *p*s<.001) with the standardized depression and anxiety raw scores across both samples.

**Figure 1:**
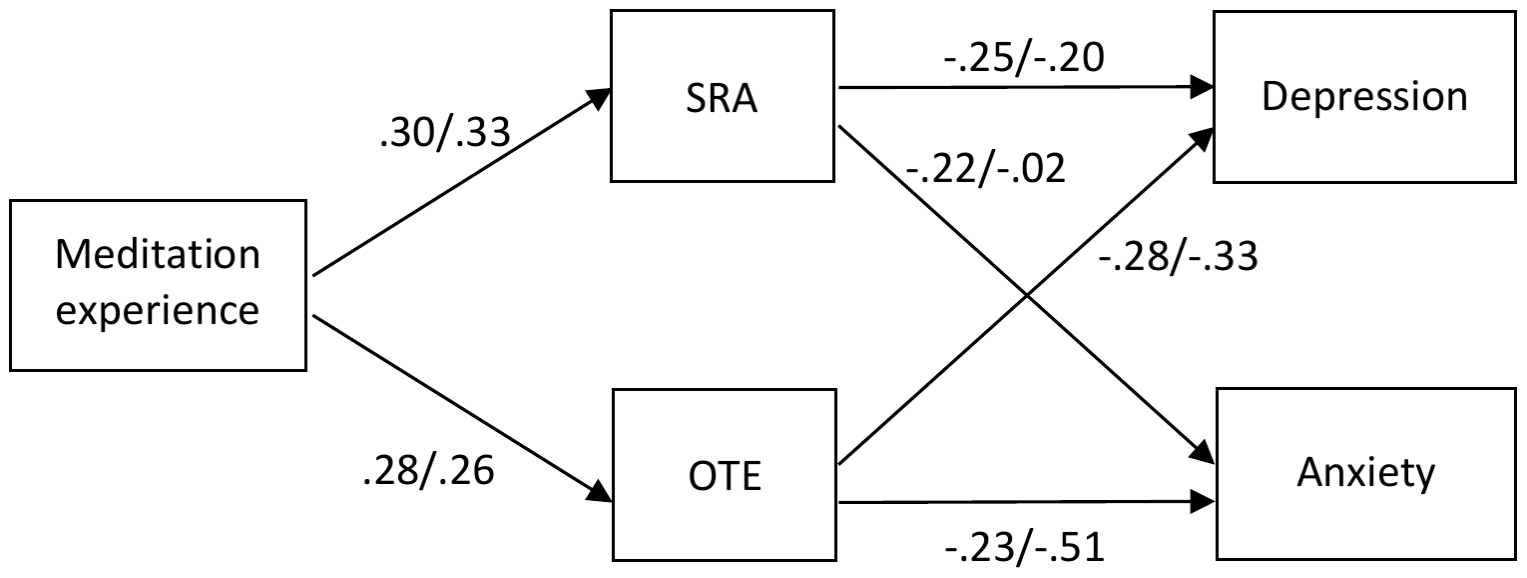
Multigroup path model on the effects of meditation experience on mindfulness and on depression and anxiety. Numbers are standardized path coefficients (left: German sample; right: Spanish sample). SRA  =  Self-regulated Attention; OTE  =  Orientation to Experience. Self-regulated Attention and Orientation to experience were allowed to correlate, as were depression and anxiety. All *p*s <.002, except for the path of SRA to anxiety in the Spanish sample, *p*  = .786.

The multigroup model, depicted in Figure 1, had a good fit to the data, χ^2^(4)  = 2.11, *p*  = .716, CFI  =  1.000, TLI  = 1.000, RMSEA  = .000 [.000,.044]. There was an overall tendency that Self-regulated Attention was slightly less indicative of depression and anxiety than Orientation to Experience. However, there were also substantive differences between the two samples: Effects of Orientation to Experience on anxiety were markedly higher among the Spanish than the German meditators. In turn, the contribution of Self-regulated Attention on anxiety was negligible among Spanish meditators, but of similar size to that of Orientation to Experience among the German meditators. Overall, the model explained 24% (24%) of the variance of depression scores in German and Spanish meditators, respectively, and 18% (27%) of anxiety scores. The total effect (standardized estimates) of meditation experience on depression and anxiety scores amounted to -.15/-.15 (depression), and -.13/-.14 (anxiety; all *p*s <.001) in German and Spanish meditators, respectively.

#### Perceived stress

**Figure 2:**
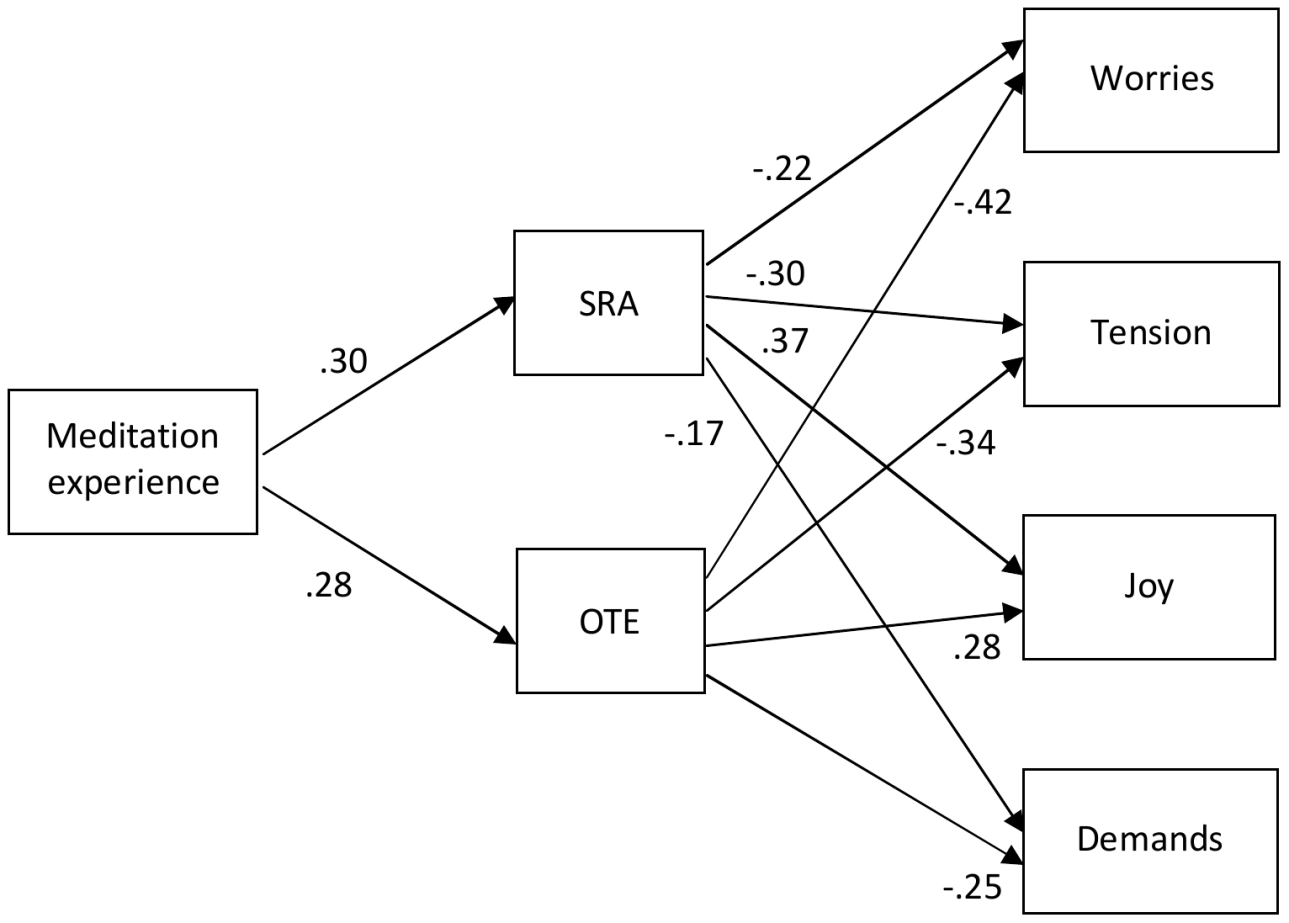
Path model on the effects of meditation experience on mindfulness and on facets of perceived stress in the German data. Numbers are standardized path coefficients. SRA  =  Self-regulated Attention; OTE  =  Orientation to Experience. Self-regulated Attention and Orientation to experience were allowed to correlate, as were the facets of perceived stress. All *p*s≤.001.

German meditators were similar in their reported levels of perceived stress (Table S3) to healthy adults [43]. A similar path model as for depression and anxiety was fitted to the German data regarding perceived stress (Table S3). The model fitted the data well, χ^2^(4)  =  5.95, *p*  = .203, CFI  = .999, TLI  = .997, RMSEA  = .023 [.000,.060], and is depicted in Figure 2. Orientation to Experience was, again, a slightly stronger predictor of all facets of perceived stress save Joy than Self-regulated Attention, corroborating the pattern obtained before with regard to depression and anxiety. Overall, the model explained 36% to 38% of the variance of Worries, Tension, and Joy scores, respectively, and 16% of the Demands score variance. The total effect (standardized estimates) of meditation experience on the former three scores was around.18 each (*p*s <.001) and.12 (*p* <.001) on Demands scores.

### Mechanisms of mindfulness

Two final path models were fitted on the Spanish data (Table S4) to explore (1) mechanisms of mindfulness and (2) to identify those mechanisms that exerted unique beneficial effects on mental health. The first path model incorporated paths from meditation experience to the higher-order factors of mindfulness that, in turn, had paths to all proposed mechanisms. Paths of the higher-order factors to mechanisms were then deleted, where insignificant (*p*>.05). The final model had a good fit to the data, χ^2^(18)  =  35.42, *p*<.001, CFI  = .993, TLI  = .960, RMSEA  = .050 [.025,.074]. Standardized path coefficients, explained variance, and total effects of meditation experience on mechanisms are presented in Table 7. Mindfulness was associated with all proposed mechanisms, strongest with Decentering and Nonattachment. Mindfulness was weakest associated with Emotional Awareness and Activation Control. Notably, a number of mechanisms were differentially associated with the higher-order factors of mindfulness: Acceptance of Emotions, Control & Regulation, Emotional Clarity, Attentional Control, Goals, Bodily Association, and Activation Control were stronger or exclusively associated with Orientation to Experience, whereas Body Awareness, Inhibitory Control,and Emotional Awareness with Self-regulated Attention. For Decentering and Nonattachment associations with the higher-order factors were of similar magnitude.

**Table 7:** Mechanisms of Mindfulness in Descending Order of Explained Variance.

	Standardized path coefficients			Total effect of meditation
	SRA	OTE	*R* ^2^	experience
Decentering	.45	.40	61%	.25
Nonattachment	.39	.36	47%	.22
Acceptance of Emotions^a^	.21	.46	38%	.19
Control & Regulation^a^	.25	.41	37%	.19
Emotional Clarity^a^	.23	.40	35%	.18
Attentional Control	–	.59	35%	.15
Body Awareness	.52	–	27%	.17
Inhibitory Control	.35	.21	26%	.17
Goals^a^	–	.51	26%	.14
Body Association^b^	–	.33	11%	.09
Emotional Awareness^a^	.33	–	10%	.10
Activation Control	–	.32	10%	.08

*Note*. Results of a path model on the effects of meditation experience on mindfulness and mechanisms of mindfulness in the Spanish data. SRA  =  Self-regulated Attention; OTE  =  Orientation to Experience. ^a^ DERS scales were reverse scored so that higher scores reflected greater ability, not more problems with emotion regulation. ^b^ The Body Dissociation scale was reverse scored so that higher scores reflected bodily association. All *p*s<.001, except the path of SRA on Control & Regulation, *p* = .002.

**Figure 3:**
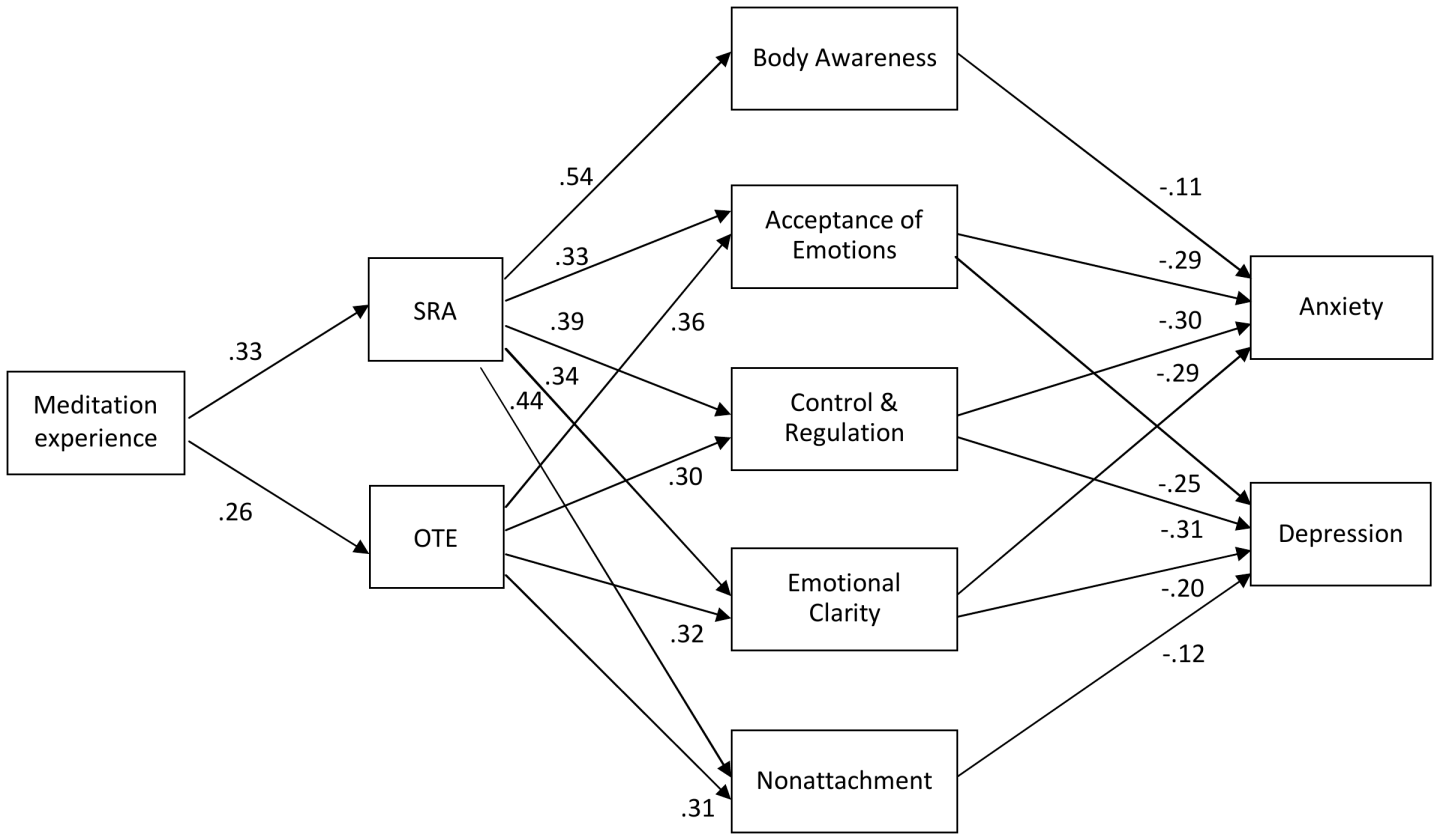
Path model on the effects of meditation experience on mindfulness, mechanisms of mindfulness, and depression and anxiety in the Spanish data. Numbers are standardized path coefficients. SRA  =  Self-regulated Attention; OTE  =  Orientation to Experience. Self-regulated Attention and Orientation to experience were allowed to correlate, as were mechanisms, and depression and anxiety. All *p*s <.015.

In the second model, depression and anxiety were included as endogenous variables, connected with paths to the mechanisms that were now mediating variables. Paths of mechanisms to depression and anxiety that were insignificant (*p*>.05), and mechanisms themselves that had no remaining significant paths, were then deleted in a stepwise procedure. The final model had a good fit to the data, χ^2^(16)  =  10.87, *p*  = .818, CFI  =  1.000, TLI  =  1.000, RMSEA  = .000 [.000,.029], it is depicted in Figure 3. Body Awareness, Acceptance of Emotions, Control & Regulation, Emotional Clarity, and Nonattachment remained in the model. For both depression and anxiety, Acceptance of Emotions, Control & Regulation, and Emotional Clarity were important mechanisms, Acceptance of Emotions most important regarding depression, Emotional Clarity most important regarding anxiety. Otherwise, Nonattachment was a further important and specific mechanism regarding depression, whereas Body Awareness regarding anxiety. Overall, the model explained 59% of the variance of depression scores and 57% of the variance of anxiety scores. The total effect (standardized estimates) of the higher-order factors (Self-regulated Attention/Orientation to Experience) on depression and anxiety scores amounted to -.33/-.31 (depression) and -.26/-.32 (anxiety; all *p*s <.003).

## Discussion

In two large and independent samples of German and Spanish meditators we investigated the psychometric and structural properties of the FFMQ, constructed a short form of the FFMQ, and confirmed a two-factor higher-order structure of mindfulness, delineating Self-regulated Attention and Orientation to Experience, previously reported in samples of non-meditators [20]. The higher-order factors contributed mostly similarly to mental health and, among the proposed mechanisms of action, fostered strongest emotion regulation and a detached, but adaptive, perspective on thoughts, emotions, and the self. Body and emotional awareness, and inhibitory control (i.e., control over responses that entail approach behavior) had unique, or stronger, paths to Self-regulated Attention, whereas bodily association, and attentional and activation control (i.e., the intentional management of attention and control over responses that entail avoidance behavior) to Orientation to Experience. Body awareness, a detachment from identifying with a static self, and, especially, the accepting and regulating aspects of emotion regulation were found to be mediators that uniquely explained the associations of meditation and mindfulness with depression and anxiety. We discuss our findings in detail in the following.

### FFMQ short form

Our item selection in meditator samples broadly reproduced the previous item selection in non-meditator samples [20]. Item selections were identical, or mostly identical, in Observe, Actaware, and Nonjudge, but differed in Describe and, even more so, in Nonreact. Shortening the FFMQ raised model fit and improved psychometric properties, as in previous research with non-meditators [20]. Differences in item selections may be explained by differences between meditators and non-meditators in comprehending the item contents of the FFMQ [7]. Van Dam et al. [7] reported that non-meditators, but not meditators, responded differently to positively and negatively worded items. Nonreact had only weak psychometric properties in non-meditating samples [14], [20], but it was the most salient facet of Self-regulated Attention in the meditator samples in the present study, surpassing Observe, that proved the most salient facet in non-meditating samples [20]. Nonreact may thus measure different constructs in meditating and non-meditating samples. It has been previously suggested [58] that among meditator samples Observe and Nonreact could be especially sensitive to formal meditation practice [25], [59]. Considering this, both facets, along with decentering, seem to efficiently differentiate among those who have daily practice meditation from those who never have meditated [2], [59].

Given the seemingly paradoxical positive associations of Observe with symptoms of anxiety in non-meditating samples in previous research [14], [20], [25], it is of interest that the selection of Observe items among meditators here was fully consistent with the previous item selection among non-meditators; i.e., even though Observe differs with regard to its effects among meditators and non-meditators, items appear to measure one-and-the-same construct.

In conclusion, the previous FFMQ item selection obtained in non-meditating samples [20] was mostly replicated in the meditating samples in the present study, suggesting that the previously proposed short form is mostly also valid for meditators, and this not only in German language, but also in Spanish. With regard to Nonreact, it is recommended to use the full scale in future assessments with meditators and non-meditators, as item selections, psychometric properties, and possibly also measured trait, differed for this scale between meditators and non-meditators.

### Higher-order structure of mindfulness

Replicating previous results [20], a two-factor higher-order structure of mindfulness was recovered in the meditating samples in the present study, with very similar results among German and Spanish meditators. As predicted, the higher-order factors correlated higher among meditators, *r*  = .69 and.62 in the German and Spanish samples, than among non-meditators, *r*  = .18 to.27 [20]. These results may be interpreted as a direct proof, and suggest a broad applicability, of the two-component model of mindfulness [3], with regard to both the conceptualization and measurement of mindfulness in the domain of self-report. In essence, these results suggest that self-reported mindfulness is, both among meditators and non-meditators, a multi-facetted, but two-factorial construct, whose homogeneity increases with meditation experience. The two-component model is also informative for, readily compatible with, and applicable to, traditional Buddhist and contemporary meditation practices, and has also received broad neuroscientific support [7]. We thus recommend using such a two-component model as the standard model to describe and explain mindfulness.

The observed differences between Spanish and German meditators regarding mean levels in Orientation to Experience could stem from sample differences: First, the Spanish sample comprised relatively more Vipassana and Zen practitioners than the German sample. Vipassana and Zen meditation does not involve much physical motion, whereas yoga, of which the German sample included a high percentage of practitioners, has a focus on bodily movements, using postures, or *asanas*, and slow movements to center the attention, which may help training Orientation to Experience more than Vipassana or Zen.

Second, the German meditators had been practicing longer, and were thus in all likelihood more experienced, than the Spanish meditators. Previous research suggests increases of mindfulness with practice [2], [22], [25], [59], [60]. Taken at face value, our results suggest that Orientation to Experience increased at a possibly slower rate than Self-regulated Attention, or, alternatively, with a time-lag, as Self-regulated Attention did not differ between the two samples. This would fit conceptual and neuroscientific considerations on meditation practice: Apparently, some training in attention regulation is needed to proceed to Orientation to Experience [6], [7]. However, statistically, it may not be ruled out that age, or cohort effects, were fully or partially responsible for the observed associations of meditation practice and mindfulness [22]. Effects of meditation style and meditation experience should be investigated in more detail in future research, using longitudinal study designs.

### Effects of mindfulness and meditation on mental health

Facets of mindfulness showed negative associations with anxiety and depression in both samples, similar to previous studies with meditating samples [14], [25], but see [23], and also with perceived stress. Apparently (neglecting the limitations of the cross-sectional design of the present study; see above), mindfulness fully mediated the effects of meditation practice with regard to depression and anxiety [25], and with perceived stress. Orientation to Experience and Self-regulated Attention predicted anxiety, depression, and perceived stress. While the mediating effects of the two-higher factors were also similar in magnitude among the German meditators, only Orientation to Experience, but not Self-regulated Attention predicted anxiety among the Spanish meditators. This latter result compares to findings in non-meditating samples, where Orientation to Experience, but not Self-regulated Attention, showed associations with mental health [20]. This difference may be interpreted as a further indication of mindfulness being a more homogeneous construct among meditators than non-meditators, not only with regard to its latent structure (see above), but also with regard to its effects. Moreover, it suggests also differences between more and less experienced meditators and/or, alternatively, between different meditation styles.

### Mechanisms of mindfulness

Decentering appeared most of all mechanisms related to meditation practice and was to the largest extent explained by the higher-order factors of mindfulness. Decentering, or metacognitive awareness, is considered a core product of mindfulness practice [2], [52] and one of the main objectives of Mindfulness-Based Cognitive Therapy [61]. We obtained empirical evidence that decentering shows a substantial overlap with mindfulness and may thus be considered one of its core mechanisms in experienced meditators.

Nonattachment was found another important mechanism that, however, had not yet received broad attention [6]. Nonattachment may not only be a consequence of practice, but it is also a core concept of Buddhism. In the present study, both samples included, with high percentages, Buddhist meditators, which could have affected the obtained results. Further research is needed to investigate whether nonattachment is a general consequence of meditation practice and training, and a general correlate of mindfulness, or whether this association depends on a specific Buddhist background of meditation practice.

Even though decentering and nonattachment were mechanisms of action that were most closely associated with mindfulness, it was mostly *other* mechanisms, or in the case of decentering, more specific mechanisms, that were found to uniquely explain the beneficial effects of mindfulness and meditation on mental health. Consistent with neuroscientific evidence [7], aspects of emotion regulation, i.e., the accepting and nonjudgmental attitude towards one's emotions, emotional clarity, and the capacity to control and regulate emotional reactions, appeared in this study the most important mechanisms of action with regard to symptoms of both depression and anxiety.

Bodily awareness appeared to convey a specific anxiolytic effect, whereas nonattachment a specific antidepressant effect. Even though these effects were rather small, these findings corroborate that mindfulness affects symptoms of depression and anxiety via different pathways [37]. As predicted previously [20], it appears that the exposure to bodily sensory information in meditation gradually brings about a change in how this information is processed: Meditators in this study obviously interpreted bodily sensory information less as a sign of anxious arousal compared to non-meditators who, in contrast, show ruminative tendencies and positive associations of Observe with anxiety [14], [20], [25]. Besides setting a focus on emotion regulation, MBIs for anxiety disorders may thus benefit from including elements that specifically focus on bodily awareness. Vice versa, one may further expect that interventions, which already include a focus on bodily awareness, increase also mindfulness, regardless of their theoretical basis. This needs to be followed up in future research.

The potential of nonattachment for a specific antidepressant effect, as observed in the present study, appears to be a new finding (but see also [51]). Buddhist thought (for a Western perspective see, e.g., [62], [63]) highlights that attachment (i.e., the mental fixation on ideas, images, or sensory objects, combined with an internal pressure to get, hold, avoid, or change circumstances or experiences) is a constant source of suffering. Nonattachment may facilitate “to ‘let go’ of psychological strategies that unintentionally promote or prolong suffering” [51] (p. 121). Nonattachment needs to be investigated in more detail in future studies. Its specific effects in MBIs for the treatment of depression need to be more closely examined. It should also be checked whether its effects depend on a Buddhist background of mediation (see above).

Different aspects of attention control were found to be differently associated with Self-regulated Attention and Orientation to Experience. This result appears interesting, as the two-component model of mindfulness [3] remains somewhat ambiguous with regard to attention control. Our results suggest that the voluntary allocation of attention (attentional control) and the capacity to control responses that entail avoidance behavior (activation control) are mechanisms of Orientation to Experience, whereas the capacity to control responses that entail approach behavior (inhibitory control) is more strongly governed by Self-regulated Attention, corroborating the specific assumptions of the two-component model [3] in this regard. However, more research is needed here, especially with regard to approach and avoidance behaviors that are implied by these different attentional processes.

### Limitations

The Spanish meditator sample was recruited through the Internet, which could have resulted in a selection bias regarding the underrepresentation of specific groups of persons or meditation types (e.g., Tibetan or yoga meditation that were much more frequent in the German sample). Generally, it is not assured that the proportions of meditation styles as observed in the present study, or the sociodemographic characteristics of the participants, were in any way representative of meditators in German-speaking countries or Spain, as participation was wholly self-assigned.

Given the cross-sectional design of the study, causal inferences were not possible. In principle, assumed effects of meditation experience could have been caused, partially or fully, by age or cohort effects that could not be disentangled, and examined directly, in the present study.

All analyses reported in the present study relied on self-report data. Thus, results may not be generalized to other types of data and may have been affected by social desirable responding, recall bias or other forms of bias applicable to self-report data.

## Conclusions

The present study broadly confirmed a previous item selection for a short form of the FFMQ and replicated a higher-order structure with two factors, Orientation to Experience and Self-regulation Attention, in samples of experienced German and Spanish meditators. The obtained results may be regarded as empirical validation of the two-component model of mindfulness [3], for which direct evidence among meditators was previously lacking. Adopting a two-component conceptualization for the FFMQ, and for the study of the mindfulness as a psychological construct, is thus recommended for future research. This may specifically benefit research on mindfulness-based interventions, but also on the psychological mechanisms underlying mindfulness practice and meditation. Mechanisms of bodily awareness and nonattachment need to be further investigated in the future with regard to their specific effects on symptoms of anxiety and depression.

## Supporting Information

Table S1 **Factor Loadings in the Five-Factor Multigroup ESEM Analysis.**
(DOCX)

Table S2 **Correlations of FFMQ Factor Scores (Short Form) with Meditation Experience, Depression, and Anxiety in Both Samples, and Means and Standard Deviations of Factor Scores and of Depression and Anxiety, Differentiated By Sample.**
(DOCX)

Table S3 **Correlations of Perceived Stress (PSQ) with Meditation Experience and Higher-Order Factor Scores of Mindfulness, and Means and Standard Deviations in the German Sample.**
(DOCX)

Table S4 **Correlations of Proposed Mechanisms of Mindfulness with Meditation Experience, Higher-Order Factor Scores of Mindfulness, Depression, and Anxiety, and Means and Standard Deviations in the Spanish Sample.**
(DOCX)
