# Supplementary material for: The Serenity of the Meditating Mind: A Cross-Cultural Psychometric Study on a Two-Factor Higher Order Structure of Mindfulness, Its Effects, and Mechanisms Related to Mental Health among Experienced Meditators
Source: PLoS One. 2014 Oct 16;9(10):e110192. doi: 10.1371/journal.pone.0110192 (PMC4199716; doi:10.1371/journal.pone.0110192)
Supplement: Table S1 — Factor Loadings in the Five-Factor Multigroup ESEM Analysis. (DOCX) [file pone.0110192.s001.docx]

**Table S1**

*Factor Loadings in the Five-Factor Multigroup ESEM Analysis*

| Item | Observe | Describe | Actaware | Nonjudge | Nonreact |
| --- | --- | --- | --- | --- | --- |
| 1 | **.50/.43** | -.04/-.03 | -.09/-.08 | .03/.03 | .28/.24 |
| 2 | .06/.06 | **.83/.82** | .05/.05 | .06/.06 | .11/.13 |
| 3 | .07/.07 | .01/.01 | .05/.06 | **.69/.68** | -.07/-.07 |
| 4 | .01/.01 | .00/.00 | .01/.01 | -.03/-.03 | **.65/.72** |
| 5 | .03/.03 | .06/.05 | **.81/.85** | -.09/-.09 | -.16/-.16 |
| 6 | **.51/.54** | .01/.01 | -.12/-.13 | .00/.00 | .13/.14 |
| 7 | -.01/-.01 | **.76/.76** | .02/.02 | .03/.04 | .13/.15 |
| 8 | -.10/-.11 | .00/.00 | **.64/.70** | .10/.10 | -.01/-.01 |
| 9 | .14/.11 | .06/.04 | -.07/-.06 | -.01/-.01 | **.63/.53** |
| 10 | -.04/-.04 | .02/.02 | .06/.07 | **.78/.79** | -.01/-.01 |
| 11 | **.42/.41** | .02/.01 | .06/.06 | .02/.02 | .12/.11 |
| 12 | .17/.20 | **-.81/-.82** | .17/.20 | .04/.04 | .01/.02 |
| 13 | .11/.12 | -.02/-.01 | **.85/.93** | -.10/-.11 | -.21/-.22 |
| 14 | .02/.02 | -.16/-.14 | .02/.02 | **.78/.81** | -.01/-.01 |
| 15 | **.72/.68** | .02/.02 | -.02/-.02 | -.05/-.04 | .15/.14 |
| 16 | .18/.21 | **-.84/-.86** | .18/.21 | .07/.08 | .01/.01 |
| 17 | .07/.08 | .02/.02 | .01/.01 | **.75/.85** | -.02/-.02 |
| 18 | -.04/-.05 | -.01/-.01 | **.59/.73** | .04/.04 | -.14/-.17 |
| 19 | .08/.08 | .04/.03 | .02/.02 | -.07/-.07 | **.67/.72** |
| 20 | **.55/.70** | -.07/-.08 | -.02/-.02 | .02/.02 | .07/.09 |
| 21 | .11/.10 | .06/.05 | -.09/-.09 | -.09/-.08 | **.53/.50** |
| 22 | -.01/-.01 | **-.64/-.62** | .16/.19 | .11/.12 | .06/.06 |
| 23 | -.33/-.30 | -.09/-.07 | **.53/.50** | .05/.05 | .12/.11 |
| 24 | .00/.00 | .02/.02 | -.06/-.06 | -.08/-.08 | **.66/.68** |
| 25 | .00/.00 | .00/.00 | -.02/-.02 | **.90/.92** | .00/.00 |
| 26 | **.72/.68** | .06/.05 | -.01/-.01 | -.05/-.05 | .03/.03 |
| 27 | .04/.05 | **.64/.65** | .00/.01 | -.03/-.03 | .15/.18 |
| 28 | -.23/-.24 | -.02/-.02 | **.56/.62** | .09/.10 | -.07/-.08 |
| 29 | -.03/-.02 | -.01/-.01 | .05/.05 | -.07/-.06 | **.83/.78** |
| 30 | -.05/-.05 | -.05/-.04 | -.06/-.06 | **.90/.89** | -.01/-.01 |
| 31 | **.63/.57** | .12/.10 | .03/.03 | -.05/-.04 | .03/.03 |
| 32 | .16/.14 | **.72/.54** | .11/.10 | .09/.07 | -.05/-.04 |
| 33 | .01/.01 | -.01/-.01 | -.01/-.01 | -.09/-.08 | **.80/.78** |
| 34 | -.37/-.37 | .00/.00 | **.72/.76** | .04/.04 | .08/.08 |
| 35 | -.02/-.02 | .00/.00 | .04/.04 | **.74/.74** | -.09/-.09 |
| 36 | **.28/.27** | .22/.19 | -.02/-.02 | .19/.18 | .41/.39 |
| 37 | .10/.11 | **.73/.70** | .03/.03 | .06/.07 | .17/.19 |
| 38 | -.23/-.28 | .00/.00 | **.53/.68** | .00/.00 | .06/.08 |
| 39 | -.08/-.09 | -.01/-.01 | .00/.00 | **.76/.86** | -.01/-.01 |

*Note.* Numbers refer to standardized factor loadings (left: German sample; right: Spanish sample). Boldface marks items that load on the designated factor. Italics highlight cross-loadings with a size of ≥ .20 in at least one of the two samples. Significance of loadings (*p* < .05) was attained for loadings of absolute size around ≥ .05.
