# Supplementary material for: The Serenity of the Meditating Mind: A Cross-Cultural Psychometric Study on a Two-Factor Higher Order Structure of Mindfulness, Its Effects, and Mechanisms Related to Mental Health among Experienced Meditators
Source: PLoS One. 2014 Oct 16;9(10):e110192. doi: 10.1371/journal.pone.0110192 (PMC4199716; doi:10.1371/journal.pone.0110192)
Supplement: Table S2 — Correlations of FFMQ Factor Scores (Short Form) with Meditation Experience, Depression, and Anxiety in Both Samples, and Means and Standard Deviations of Factor Scores and of Depression and Anxiety, Differentiated By Sample. (DOCX) [file pone.0110192.s002.docx]

**Table S2**

*Correlations of FFMQ Factor Scores (Short Form) with Meditation Experience, Depression, and Anxiety in Both Samples, and Means and Standard Deviations of Factor Scores and of Depression and Anxiety, Differentiated By Sample*

|  | Correlation with | | | *M* (*SD*) |  |
| --- | --- | --- | --- | --- | --- |
|  | Med. exp.^a^ | Depression^b^ | Anxiety^b^ | German | Spanish |
| Observe | .20 | -.30 | -.30 | 0.17 (0.93) | -0.01 (0.87) |
| Describe | .18 | -.31 | -.26 | 0.02 (0.87) | -0.02 (0.84) |
| Actaware | .28 | -.40 | -.41 | 0.42 (0.89) | 0.00 (0.85) |
| Nonjudge | .24 | -.42 | -.41 | 0.18 (0.90) | -0.04 (0.86) |
| Nonreact | .29 | -.45 | -.44 | -0.08 (0.98) | 0.00 (0.86) |
| SRA | .29 | -.46 | -.45 | -0.07 (1.10) | 0.00 (0.97) |
| OTE | .33 | -.45 | -.46 | 0.55 (0.95) | 0.00 (0.90) |
| Depression^b^ | -.11 | – | .58 | 0.46 (0.54) | 6.14 (7.70) |
| Anxiety^b^ | -.16 | .58 | – | 0.49 (0.51) | 5.14 (6.27) |

*Note*. Med. exp. = mediation experience; SRA = Self-regulated Attention; OTE = Orientation to Experience. ^a^ Log-months were used for correlational analyses (*n* = 1086 due to incomplete data). ^b^ Measured with the BSI in the German sample and the DAS-21 in the Spanish sample; standardized scores were used for correlational analyses, means and standard deviations refer to raw scores (*n* = 367 in the Spanish sample due to incomplete data). For correlation coefficients, all *p*s < .001.
