# Supplementary material for: The Serenity of the Meditating Mind: A Cross-Cultural Psychometric Study on a Two-Factor Higher Order Structure of Mindfulness, Its Effects, and Mechanisms Related to Mental Health among Experienced Meditators
Source: PLoS One. 2014 Oct 16;9(10):e110192. doi: 10.1371/journal.pone.0110192 (PMC4199716; doi:10.1371/journal.pone.0110192)
Supplement: Table S3 — Correlations of Perceived Stress (PSQ) with Meditation Experience and Higher-Order Factor Scores of Mindfulness, and Means and Standard Deviations in the German Sample. (DOCX) [file pone.0110192.s003.docx]

**Table S3**

*Correlations of Perceived Stress (PSQ) with Meditation Experience and Higher-Order Factor Scores of Mindfulness, and Means and Standard Deviations in the German Sample*

|  | Correlation with | | |  |
| --- | --- | --- | --- | --- |
|  | Med. exp.^a^ | SRA | OTE | *M* (*SD*) |
| Worries | -.16 | -.54 | -.59 | 0.24 (0.22) |
| Tension | -.19 | -.56 | -.57 | 0.32 (0.22) |
| Joy | .16 | .59 | .57 | 0.65 (0.23) |
| Demands | -.07 | -.37 | -.39 | 0.36 (0.23) |

*Note*. Med. exp. = mediation experience; SRA = Self-regulated Attention; OTE = Orientation to Experience. ^a^ Log-months were used for correlational analyses (*n* = 738 due to incomplete data). For correlation coefficients, all *p*s < .001, except for meditation experience with Joy, *p* = .045.
