# Supplementary material for: The Serenity of the Meditating Mind: A Cross-Cultural Psychometric Study on a Two-Factor Higher Order Structure of Mindfulness, Its Effects, and Mechanisms Related to Mental Health among Experienced Meditators
Source: PLoS One. 2014 Oct 16;9(10):e110192. doi: 10.1371/journal.pone.0110192 (PMC4199716; doi:10.1371/journal.pone.0110192)
Supplement: Table S4 — Correlations of Proposed Mechanisms of Mindfulness with Meditation Experience, Higher-Order Factor Scores of Mindfulness, Depression, and Anxiety, and Means and Standard Deviations in the Spanish Sample. (DOCX) [file pone.0110192.s004.docx]

**Table S4**

*Correlations of Proposed Mechanisms of Mindfulness with Meditation Experience, Higher-Order Factor Scores of Mindfulness, Depression, and Anxiety, and Means and Standard Deviations in the Spanish Sample*

|  | Correlation with | |  | | |  | |  |
| --- | --- | --- | --- | --- | --- | --- | --- | --- |
|  | Med. exp.^a^ | SRA | | OTE | Depression | | Anxiety | *M* (*SD*) |
| Attentional Control | .20*** | .44*** | | .58*** | -.39*** | | -.34*** | 23.20 (5.10) |
| Inhibitory Control | .14* | .50*** | | .45*** | -.41*** | | -.37*** | 35.79 (5.86) |
| Activation Control | .11 | .25*** | | .32*** | -.34*** | | -.24*** | 34.97 (5.57) |
| Body Awareness | .13* | .54*** | | .44*** | -.32*** | | -.19*** | 3.76 (0.52) |
| Body Association^b^ | .04 | .30*** | | .35*** | -.37*** | | -.40*** | 2.02 (0.51) |
| Decentering | .27*** | .73*** | | .72*** | -.55*** | | -.51*** | 41.05 (6.08) |
| Acceptance of Emotions^c^ | .11* | .57*** | | .58*** | -.70*** | | -.68*** | 11.08 (5.32) |
| Goals^c^ | .17** | .48*** | | .50*** | -.60*** | | -.54*** | 8.27 (3.17) |
| Control & Regulation^c^ | .16** | .59*** | | .56*** | -.72*** | | -.69*** | 13.13 (5.64) |
| Emotional Awareness^c^ | .00 | .31*** | | .25*** | -.17*** | | -.19*** | 13.86 (1.80) |
| Emotional Clarity^c^ | .14** | .56*** | | .57*** | -.63*** | | -.63*** | 6.36 (2.40) |
| Nonattachment | .19** | .65*** | | .63*** | -.61*** | | -.51*** | 4.94 (0.71) |

*Note*. Med. exp. = mediation experience; SRA = Self-regulated Attention; OTE = Orientation to Experience. Sample size with regard to means and standard deviations varied from *n* = 340 (Body Awareness and Body Association) to *n* = 360 (DERS scales) due to incomplete data; sample size of correlational analyses varied from *n* = 302 to *n* = 360 due to incomplete data also in meditation experience (*n* = 348) and depression and anxiety scores (*n* = 368). ^a^ Log-months were used for correlational analyses. ^b^ The Body Dissociation scale was reverse scored for correlation analyses so that higher scores reflected bodily association. ^c^ DERS scales were reverse scored for correlation analyses so that higher scores reflected greater ability, not more problems with emotion regulation. * *p* < .05, ** *p* < .01, *** *p* < .001.
